# Is There an Association Between Fixed Orthodontic Treatment and Initiation of Eating Disorders? A Review of Currently Available Evidence

**DOI:** 10.3389/froh.2021.707040

**Published:** 2021-07-12

**Authors:** Melina Koukou, Fawad Javed, Dimitrios Michelogiannakis

**Affiliations:** ^1^Faculty of Dentistry, National and Kapodistrian University of Athens, Athens, Greece; ^2^Department of Orthodontics and Dentofacial Orthopedics, Eastman Institute for Oral Health, University of Rochester, New York, NY, United States

**Keywords:** orthodontic therapy, eating disorders, anorexia nervosa, bulimia, review

## Abstract

**Objectives:** The aim was to review the available literature regarding the potential association between fixed orthodontic treatment (OT) and the onset of eating disorders (EDs).

**Method and Materials:** Six indexed databases were searched until November 2020. The inclusion criteria were as follows: (a) patients undergoing fixed OT and (b) EDs in relation to fixed OT. Commentaries, letters to the Editor, reviews, and studies in patients with EDs not undergoing fixed OT were excluded. The pattern of the present review was customized to summarize the pertinent information.

**Results:** Four out of 10,076 initially-identified studies were included, and all of them were case reports. All patients were females, and the EDs reported were either anorexia nervosa (AN) or bulimia nervosa (BN). In three case reports, patients developed EDs after the initiation of OT. Fixed OT was performed in all the studies, and a variety of oral complications such as sore mouth, gingivitis, tooth surface demineralization, and others were reported.

**Conclusion:** Based upon the limited available evidence, the association between OT and the onset of EDs remains unclear. Further well-designed observational clinical studies are needed in this regard.

## Introduction

Eating disorders (EDs) are psychiatric disorders characterized by abnormal routine eating-related behaviors [1–3], and patients often correlate them with psychological concerns related to their weight and body image [1, 2]. A mortality rate of up to 25% has been reported for patients with EDs [4–7]; and the prevalence of EDs is higher in females compared with males [4, 5, 8, 9]. The most common forms of EDs are anorexia nervosa (AN) and bulimia nervosa (BN) [7, 10, 11]. The primary characteristic of AN includes restriction of food intake due to a persistent fear of becoming overweight, even though the affected individual is underweight [1, 5, 10, 11]; whereas, BN is characterized by binge eating which, is often followed by self-induced vomiting in order to maintain a low body weight [1, 5, 6, 10, 12]. It is well-established that AN and BN are serious conditions that jeopardize the general and psychological health status of patients [4, 5]. They make their appearance more usually in adolescence, which is also the most common period during which orthodontic treatment (OT) is initiated [4, 5, 8, 9, 13]. There are a variety of oral symptoms in patients with EDs including enamel erosion, dental caries, dentinal hypersensitivity, enamel demineralization, malocclusion, and xerostomia; with tooth erosion being the most prevalent [6, 10–12, 14–16].

The OT is commonly performed for the correction of craniofacial disharmonies and dental malocclusion, and its success depends on patient compliance and regular follow-up visits [17, 18]. Moreover, oral hygiene maintenance during the course of OT is a critical factor that influences the success of planned OT as these patients are susceptible to gingival inflammation and enamel demineralization [19, 20]. It has also been suggested that psychological stress may negatively influence the outcome of planned OT in susceptible individuals [21]. Furthermore, alternations in dietary intake and weight status have been reported in patients undergoing OT [22]. Mental and physical development occurs at a rapid rate during adolescence. In addition, adolescence is a time period during which individuals are managing issues such as self-esteem and acceptance by peers [7]. It has also been reported that psychiatric disorders such as EDs may manifest during the adolescent years [7]. Due to the nature of OT, which requires regular follow-up appointments, it is postulated that the orthodontist could be the first health care provider to observe such disorders [7, 10, 11, 23]. For instance, in the study by Jaffa [24], it was reported that AN was manifested in an adolescent female after the initiation of OT. Similar results have been reported by Lee et al. [13] In this regard, the authors of the present study hypothesize that there is a potential association between OT and the onset of EDs. To the authors' knowledge, there are no studies in indexed literature that have reviewed the relationship between OT and EDs.

The aim of this study was to review the available literature regarding the potential association between fixed OT and the onset of EDs.

## Method and Materials

### Focused Question

In the present review, the Preferred Reporting Items for Systematic Reviews and Meta-Analysis guidelines [25] were followed to assess the following focused question: “Can fixed OT trigger the onset of EDs?”

### Eligibility criteria

The inclusion criteria were as follows: (a) patients undergoing fixed OT and (b) EDs in relation to fixed OT. Commentaries, letters to the Editor, reviews, and studies on patients with EDs not undergoing fixed OT were excluded. The pattern of the present review was customized to summarize the pertinent information.

### Information Sources, Search Strategy, and Study Selection

Six indexed databases [EMBASE, PubMed (National Library of Medicine), Google Scholar, ISI Web of Knowledge, OVID, and Scopus] were searched without time and language restrictions up to and including November 2020. A customized search strategy was implemented by one author (MK) (Table 1). The titles and abstracts of relevant articles were screened by two authors (MK and DM), and the full-texts of relevant articles were independently read. A hand-search of the reference lists of relevant articles was also performed to collect possible articles that may have been missed in the previous steps. Disagreements were resolved *via* discussion between three authors (MK, DM, and FJ).

**Table 1:** Keywords and database search strategy.

**Keywords**	**Search strategy**
Orthodontic; orthodontics; orthodontic therapy; orthodontic treatment; eating disorders; anorexia; anorexia nervosa; bulimia; bulimia nervosa	([orthodontic[All Fields] AND (“therapy”[Subheading] OR “therapy”[All Fields] OR “therapeutics”[MeSH Terms] OR “therapeutics”[All Fields])] OR [orthodontic[All Fields] AND (“therapy”[Subheading] OR “therapy”[All Fields] OR “treatment”[All Fields] OR “therapeutics”[MeSH Terms] OR “therapeutics”[All Fields])]) AND [(“feeding and eating disorders”[MeSH Terms] OR (“feeding”[All Fields] AND “eating”[All Fields] AND “disorders”[All Fields]) OR “feeding and eating disorders”[All Fields] OR (“eating”[All Fields] AND “disorders”[All Fields]) OR “eating disorders”[All Fields]) OR (“anorexia nervosa”[MeSH Terms] OR (“anorexia”[All Fields] AND “nervosa”[All Fields]) OR “anorexia nervosa”[All Fields]) OR (“bulimia”[MeSH Terms] OR “bulimia”[All Fields])]

### Data Collection and Data Items

Data extraction from the included studies was performed independently by two authors (MK and FJ); and pertinent information was collected as follows: (1) reference, (2) study design, (3) number of patients, (4) demographic information, (5) information regarding EDs and its treatment, (6) chronological association between EDs and OT, (7) type of malocclusion, (8) information regarding OT, (9) orofacial complications, and (10) instructions of the orthodontist. Disagreements related to data extraction were resolved through consensus discussion.

## Results

### Study Selection

**Figure 1:**
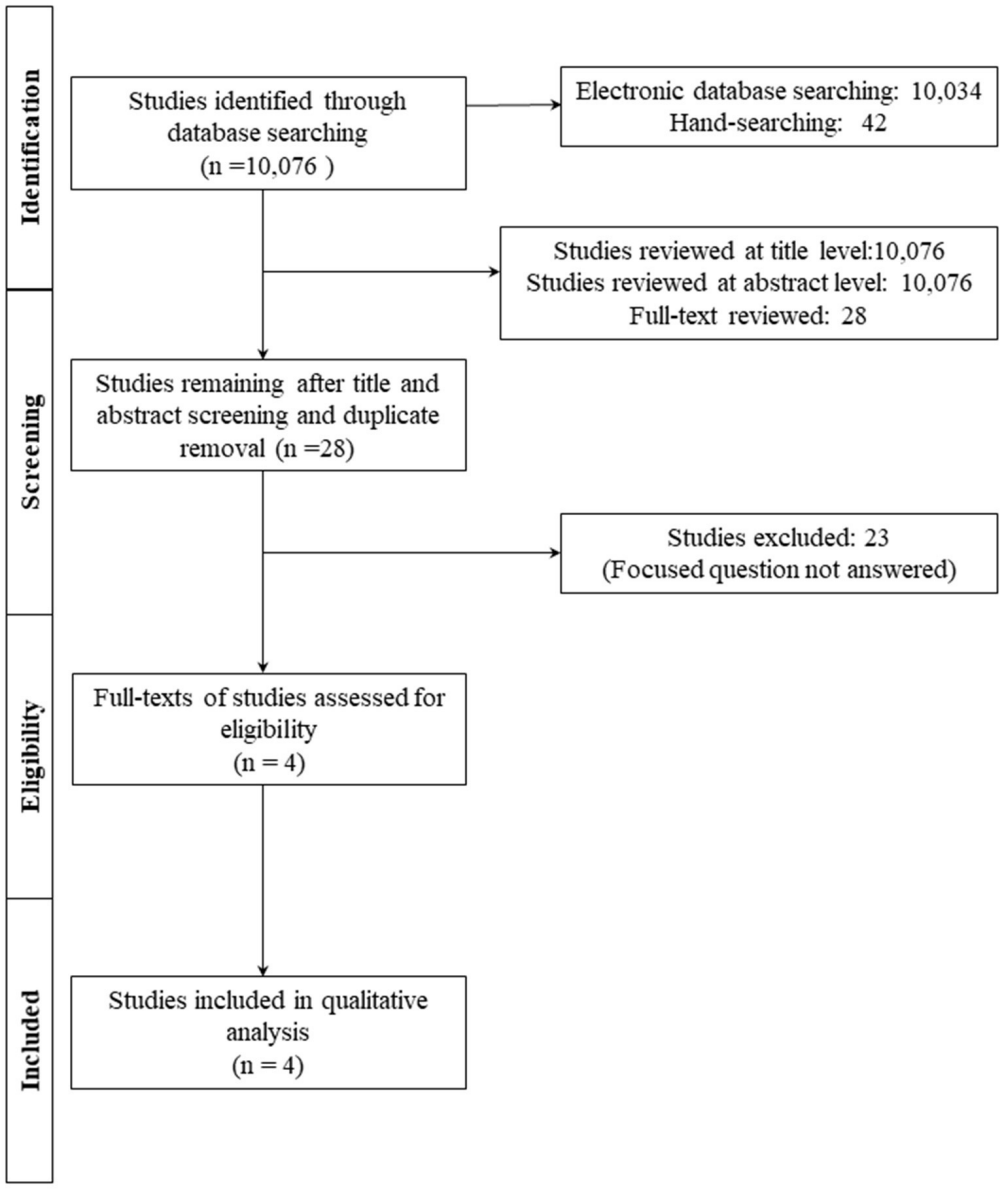
Flowchart of the study.

Initially, 10,076 studies were identified, and 10,048 studies were excluded due to irrelevant titles and abstracts or duplicate removal. Twenty-four studies were further excluded after a full-text review based on the previously-mentioned inclusion criteria. A total of four studies [6, 13, 15, 24] were included in this review and analyzed for data extraction (Figure 1, Supplementary Table A).

### General Characteristics of the Included Studies

All studies [6, 13, 15, 24] included in the present review were case reports, and the number of patients ranged between 1 and 3. In the study by Jaffa [24], a total of three patients were included; however, one patient was excluded from the present review since the patient did not undergo OT. In the three case reports [6, 13, 24], the patients were adolescents with their age ranging between 13 and 17 years. In the study by Shaw BM [15], the patient was a 30-year-old adult. All patients described were females [6, 13, 15, 24] (Table 2). These case reports were performed in the following countries: United Kingdom [24], Korea [13], France [6], and the United States [15].

**Table 2:** General characteristics of the included case reports.

**Authors (year)**	**Number of patients**	**Age**	**Gender**
Jaffa (2007) [24]	3^*^	16 years	Female
		14 years	Female
Lee et al. (2015) [13]	2	14 years	Female
		13 years	Female
Corega et al. (2014) [6]	2	13 years	Female
		17 years	Female
Shaw (1994) [15]	1	30 years	Female

* *One of the three patients did not undergo orthodontic treatment; and thus, was excluded from the present study based on the inclusion criteria*.

### Study Characteristics Related to EDs

In two studies [13, 24], patients had AN, and in two studies [6, 15], patients had BN. The onset of EDs in two studies [13, 24] was reported to be at the beginning of OT. In the study by Corega et al. [6], it was reported that the onset of EDs occurred between 4 and 7 months after the initiation of OT. In the study by Shaw BM [15], the patient had undergone OT from 9 to 11 years old, was diagnosed with BN in her teen years, and sought OT again at the age of 30 years after overcoming the ED. Sore mouth after the fitting of the braces, instructions from the orthodontist to avoid sweet foods, and comments/compliments from peers about the slim body of the patient were reported as potential triggers for the EDs in two studies [13, 24], and two studies [6, 15] did not report such potential triggers. One of the patients [24] seemed to have concerns about her body image before the ED appeared; and three patients [13, 24] did not have any such concerns. In two studies [6, 15], concerns about body image prior to ED were not reported. The diagnosis of EDs was made by a health care professional in two studies [13, 24]. The EDs resulted in the hospitalization of the patients in two studies [13, 24]. The patient in the study by Shaw BM [15] and one of the patients in Corega's report [6] were not hospitalized; while, for the other patient in the Corega's study, information about hospitalization was not reported. Treatment strategies adopted for the management of EDs were hospitalization ranging from 20 days to 4 months [13, 24], medications [13, 24], psychoeducation, supportive treatment, psychiatric treatment or psychotherapy [6, 13, 15, 24], and nutritional rehabilitation [13]. Corega et al. [6] reported two cases in which EDs were manifested in patients undergoing OT; however, in one of the patients, the treatment protocol for the ED was not reported (Table 3).

**Table 3:** Study characteristics related to the eating disorder.

**Authors (year)**	**Type of eating disorder**	**Onset of eating disorder**	**Potential trigger of abnormal eating behavior**	**Concerns with body image prior to eating disorder**	**Diagnosis of eating disorder**	**Hospitalization due to eating disorder**	**Treatment of eating disorder**
Jaffa (2007) [24]	Anorexia nervosa	At the beginning of OT	Sore mouth after fitting of braces, advice to avoid sweet foods	Yes	By healthcare provider	Yes	Inpatient psychiatric treatment and pharmacological treatment
	Anorexia nervosa	At the beginning of OT	Sore mouth after fitting of braces, comment of friend that braces result in weight lose	No	By healthcare provider	Yes	Inpatient psychiatric treatment
Lee et al. (2015) [13]	Anorexia nervosa	At the beginning of OT	Sore mouth after fitting of braces	No	By healthcare provider	Yes	Psychoeducation, supportive treatment, nutritional rehabilitation
	Anorexia nervosa	At the beginning of OT	Sore mouth after fitting of braces, comments of friends that her slim body looked better	No	By healthcare provider	Yes	Psychoeducation, psychological support, pharmacological treatment
Corega et al. (2014) [6]	Bulimia nervosa	4 months after the initiation of OT	NR	NR	NR	No	Psychiatric treatment
	Bulimia nervosa	7 months after the initiation of OT	NR	NR	NR	NR	NR
Shaw (1994) [15]	Bulimia nervosa	Years before the initiation of OT^*^	NR	NR	NR	No	Psychotherapy

*OT, orthodontic treatment; NR, not reported*.

* *Patient had a prior history of OT (9–11 years old); and bulimia nervosa was manifested during her teen years. In the present case report, the patient underwent OT again at the age of 30 years when she had overcome the eating disorder*.

### Study Characteristics Related to OT

The type of malocclusion was reported in two studies [6, 15]. Fixed appliances were used in all studies [6, 13, 15, 24]; one patient [6] was scheduled also for orthognathic surgery but OT had to be discontinued at the presurgical phase. In the report by Shaw BM [15], the interproximal surfaces of the mandibular incisors were stripped and 2 months before the fitting of the braces in the lower arch, a removable maxillary bite plane with expansion screws was used. The total duration of OT ranged between 7 and 10 months in the two studies [6, 15] that it was reported. In two studies [13, 24], it was stated that the patients were hospitalized after a period of OT ranging from 7 months to 2 years. Oral complications which were encountered in relation to EDs were sore mouth [13, 24], recurrent oral ulcers [13], generalized gingivitis [6], demineralization [6], white spot lesions [6], diffuse erythema [6], incisal-lingual enamel erosions of the upper incisors, canines and premolars, and lower incisor over-eruption [15]. The OT had to be discontinued in both the patients of the Corega's report [6], it was successfully completed in the report by Shaw BM [15], and in two studies [13, 24], the outcome of OT was not reported. Specific guidelines for the management of patients with ED by the orthodontist were not reported in any of the included studies [6, 13, 15, 24] (Table 4).

**Table 4:** Study characteristics related to orthodontic therapy.

**Authors (year)**	**Type of malocclusion**	**Type of OT**	**Total duration of OT**	**Oral complications**	**Treatment of oral complications**	**Outcome of OT**
Jaffa (2007) [24]	NR	Fixed appliances	NR^***^	Sore mouth	NR	NR
	NR	Fixed appliances	NR^***^	Sore mouth	NR	NR
Lee et al. (2015) [13]	NR	Fixed appliances	NR^****^	Sore mouth	NR	NR
	NR	Fixed appliances	NR^****^	Recurrent oral ulcers, sore mouth	NR	NR
Corega et al. (2014) [6]	Crowding	Fixed appliances	10 months	Generalized gingivitis and denineralizations, WSLs, diffuse erythema	Referral to dentist for restorative treatment	OT was discontinued
	Class II Division 2	Fixed appliances combined with planned orthognathic surgery^*^	7 months	Generalized gingivitis and denineralizations, WSLs	Referral to dentist for restorative treatment	OT was discontinued
Shaw (1994) [15]^**^	Class I, anterior closed bite, mandibular anterior crowding, narrow arches	Removable maxillary bite plane with expansion screws, stripping and fixed appliances	~8 months^**^	Incisal-lingual enamel erosions of upper incisors, canines and premolars, and lower incisor over-eruption	OT to increase interincisal space followed by placement of porcelain veneer crowns on upper incisors	Successful

*OT, orthodontic treatment; NR, not reported; WSLs, white spot lesions*.

* *The OT was discontinued at the final presurgical phase*.

** *The patient also had a prior history of OT when she was 9–11 years old*.

*** *The author reported time of hospitalization after 2 years of OT*.

**** *The authors reported time of hospitalization after 23 months of OT for the first case and after 7 months for the second*.

## Discussion

An exhaustive search of indexed literature was conducted to identify studies that addressed the focused question; however, to date, there are no cohort (prospective or retrospective) clinical studies that have assessed the association between OT and the onset of EDs. The only available evidence is in the form of case reports [6, 13, 15, 24]. In this regard, it was difficult to adopt the traditional protocols followed in systematic reviews and meta-analyses. Therefore, the pattern of the present review was customized to primarily summarize the available information.

In summary, results from 75% of the case reports [6, 13, 24] showed that OT triggered the onset of EDs in adolescent female patients. In these studies [6, 13, 24], the patients developed sore mouth after the initiation of fixed OT, and this could have influenced the routine dietary patterns of the patients. It is important to interpret these results with caution as, by no means, do the authors intend to suggest that OT is a risk factor for the onset of EDs. However, these results do suggest that OT may instigate abnormal eating behaviors in susceptible patient populations. In an age and gender-matched controlled cohort study, Shirazi et al. [22] assessed the nutritional intake of adolescents undergoing fixed OT. The results showed that adolescent patients undergoing fixed OT consumed significantly lower amounts of chromium, fiber, and beta-carotene compared with controls [22]. Similarly, Carter et al. [26] investigated the influence of fixed OT on the routine eating habits in teenagers. According to the findings of this study [26] the participants restricted food intake due to factors such as fear of breakage of orthodontic appliances, dietary advice given by their orthodontist, fear of social embarrassment, and alterations in taste perception. Moreover, in this study [26], some participants also reported that fixed OT had a significant impact on their routine dietary habits. Based upon the results reported above [22, 26], it is evident that fixed OT influences the daily eating habits of the patients; however, should these dietary alterations lead to the onset of EDs remains questionable. There is a likelihood that patients undergoing fixed OT would resume their normal eating habits following the completion of OT. However, to date, there are no cohort studies that have assessed the preoperative and postoperative eating habits of patients undergoing OT. Further, studies are needed in this regard.

There is sufficient evidence in indexed literature to confirm that oral and craniofacial health is at risk in patients diagnosed with EDs. Studies have shown that enamel erosion, parotid swellings, alterations in salivary constituents, dry lips, burning tongue, and temporomandibular disorders are more prevalent in patients with than without EDs [27, 28]. Nevertheless, the association between EDs and periodontal diseases remains debatable [28]. Moreover, according to Robinson et al. [29], patients with EDs particularly adolescent females are at increased risk of demonstrating low bone mineral density. In the case reports included [6, 13, 15, 24], oral health-related complications such as sore mouth, recurrent oral ulcers, generalized gingivitis, demineralizations, white spot lesions, diffuse erythema, and enamel erosions were manifested in all patients undergoing fixed OT. It is noteworthy, that the planned OT was successfully completed in only one case report [15]. However, based on the oral and general health-related complications, OT was discontinued in the study by Corega et al. [6]. Since EDs are a complex issue, a multidisciplinary therapeutic approach is required for the treatment of malocclusion and dentoskeletal deformities in susceptible patient groups. Such an approach may potentially include consultations with Nutritionists, Psychiatrists, Psychologists, Restorative Dentists, Dental Hygienists, and Orthodontists [30].

The risk of bias evaluation is an important aspect of a critical scientific review. Since indexed evidence available to date that addressed our focused question is solely based on case reports, the authors perceive a high risk of bias in these studies [6, 13, 15, 24]. Nevertheless, irrespective of such scientific limitations, the possibility of an existing link between OT and the onset of EDs cannot be overlooked. From an ethical aspect, patients should be informed about possible dietary and oral complications that may be encountered during the course of fixed OT. Likewise, consultations with nutritionists and psychologists for patients planned and/or scheduled to undergo fixed OT might help minimize the risk of the onset of EDs. Routine dental follow-ups in patients undergoing fixed OT may play a role in the early detection of oral complications such as tooth erosion and enamel demineralization that may be potentially induced by latent EDs. Furthermore, psychological stress is a risk factor for the onset of EDs [31] and has also been shown to influence orthodontic tooth movement [21]. The authors of the present study suggest that prescreening of potential candidates for future OT could be done using questionnaires focusing on a history of stress/anxiety disorders as well as EDs. It is, therefore, essential to educate the patients as well as health care providers about the potential bidirectional interaction between EDs and outcomes of OTs and vice versa.

### Conclusion

Based on the currently available case reports, the association between fixed OT and the onset of EDs remains unclear. Further well-designed observational clinical studies are needed in this regard.

## Data Availability Statement

The original contributions generated for the study are included in the article/Supplementary Material, further inquiries can be directed to the corresponding author/s.

## Author Contributions

FJ was responsible for conceptualization and editing. MK was responsible for writing, methodology, and data extraction. DM was responsible for writing, editing, methodology, and supervision. All authors have read and approved the final draft, contributed equally to the manuscript preparation, and made substantial contributions.

## Conflict of Interest

The authors declare that the research was conducted in the absence of any commercial or financial relationships that could be construed as a potential conflict of interest.

## References

[1] Treasure J Duarte TA Schmidt U. Eating disorders. Lancet. (2020) 395:899–911. 10.1016/S0140-6736(20)30059-3 32171414

[2] Mathis KJ Costa CB Xandre PE. Treating individuals with eating disorders: part 1. J Psychosoc Nurs Ment Health Serv. (2020) 58:7–13. 10.3928/02793695-20200217-02 32129875

[3] Wagner G Karwautz A. Eating disorders in adolescents with type 1 diabetes mellitus. Curr Opin Psychiatry. (2020) 33:602–10. 10.1097/YCO.0000000000000650 32858602

[4] Yagi T Ueda H Amitani H Asakawa A Miyawaki S Inui A. The role of ghrelin, salivary secretions, and dental care in eating disorders. Nutrients. (2012) 4:967–89. 10.3390/nu4080967 23016127 PMC3448082

[5] Alzahrani HH AlBukhari AH Mandili IM Bana RA Alzamil FO Gusti WW . Management of Eating Disorders in Primary Care. EC Microbiology. (2019) 12:01–06.

[6] Corega C Vaida L Festila DG Rigoni G Albanese M D'Agostino A . Dental white spots associated with bulimia nervosa in orthodontic patients. Minerva Stomatol. (2014). . [Epub ahead of print]. 24423744

[7] Neeley WW II Kluemper GT Hays LR. Psychiatry in orthodontics. Part 1: Typical adolescent psychiatric disorders and their relevance to orthodontic practice. Am J Orthod Dentofacial Orthop. (2006) 129:176–84. 10.1016/j.ajodo.2005.11.009 16473708

[8] Goldman SJ. Practical approaches to psychiatric issues in the orthodontic patient. Semin Orthod. (2004) 10:259–65. 10.1053/j.sodo.2004.09.006

[9] Hoek HW van Hoeken D. Review of the prevalence and incidence of eating disorders. Int J Eat Disord. (2003) 34:383–96. 10.1002/eat.10222 14566926

[10] Alqahtani H. Medically compromised patients in orthodontic practice: review of evidence and recommendations. Int Orthod. (2019) 17:776–88. 10.1016/j.ortho.2019.08.015 31471239

[11] Patel A Burden DJ Sandler J. Medical disorders and orthodontics. J Orthod. (2009) 36(Suppl. 1):1–21. 10.1179/14653120723346 19934236

[12] Bretz WA. Oral profiles of bulimic women: diagnosis and management. What is the evidence? J Evid Based Dent Pract. (2002) 2:267–72. 10.1016/S1532-3382(02)70078-X 22287937 PMC3267322

[13] Lee JY Kim SW Kim JM Shin IS Yoon JS. Two cases of eating disorders in adolescents with dental braces fitted prior to the onset of anorexia nervosa. Psychiatry Investig. (2015) 12:411–4. 10.4306/pi.2015.12.3.411 26207138 PMC4504927

[14] O'Reilly RL O'Riordan JW Greenwood AM. Orthodontic abnormalities in patients with eating disorders. Int Dent J. (1991) 41:212–6. 1917077

[15] Shaw BM. Orthodontic/prosthetic treatment of enamel erosion resulting from bulimia: a case report. J Am Dent Assoc. (1994) 125:188–90. 10.14219/jada.archive.1994.0263 8113527

[16] Faine MP. Recognition and management of eating disorders in the dental office. Dent Clin North Am. (2003) 47:395–410. 10.1016/S0011-8532(02)00108-8 12699238

[17] Li X Xu ZR Tang N Ye C Zhu XL Zhou T . Effect of intervention using a messaging app on compliance and duration of treatment in orthodontic patients. Clin Oral Investig. (2016) 20:1849–59. 10.1007/s00784-015-1662-6 26631059

[18] Klaus K Stark P Serbesis TSP Pancherz H Ruf S. Excellent versus unacceptable orthodontic results: influencing factors. Eur J Orthod. (2017) 39:615–21. 10.1093/ejo/cjx006 28371839

[19] Shah SS Nambiar S Kamath D Suman E Unnikrishnan B Desai A . Comparative evaluation of plaque inhibitory and antimicrobial efficacy of probiotic and chlorhexidine oral rinses in orthodontic patients: a randomized clinical trial. Int J Dent. (2019) 2019:1964158. 10.1155/2019/1964158 30930947 PMC6410424

[20] Benic GZ Farella M Morgan XC Viswam J Heng NC Cannon RD . Oral probiotics reduce halitosis in patients wearing orthodontic braces: a randomized, triple-blind, placebo-controlled trial. J Breath Res. (2019) 13:036010. 10.1088/1752-7163/ab1c81 31022704

[21] Al-Shammery D Michelogiannakis D Rossouw E Romanos GE Javed F. Influence of psychological stress exposure on orthodontic therapy: a comprehensive review. J Investig Clin Dent. (2019) 10:e12388. 10.1111/jicd.12388 30618117

[22] Shirazi AS Mobarhan MG Nik E Kerayechian N Ferns GA. Comparison of dietary intake between fixed orthodontic patients and control subjects. Aust Orthod J. (2011) 27:17–22. 21696109

[23] Proffit WR Fields HW Sarver DM. Contemporary Orthodontics. Mosby: Elsevier (2013).

[24] Jaffa T. Three cases illustrating the potential of dental treatment as a precipitant for weight loss leading to anorexia nervosa. Eur Eat Disord Rev. (2007) 15:42–4. 10.1002/erv.760 17676671

[25] Moher D Liberati A Tetzlaff J Altman DG. Preferred reporting items for systematic reviews and meta-analyses: the PRISMA statement. Int J Surg. (2010) 8:336–41. 10.1016/j.ijsu.2010.02.007 20171303

[26] Carter LA Geldenhuys M Moynihan PJ Slater DR Exley CE Rolland SL. The impact of orthodontic appliances on eating - young people's views and experiences. J Orthod. (2015) 42:114–22. 10.1179/1465313314Y.0000000128 26118682

[27] Frydrych AM Davies GR McDermott BM. Eating disorders and oral health: a review of the literature. Aust Dent J. (2005) 50:6–15; quiz 56. 10.1111/j.1834-7819.2005.tb00079.x 15881300

[28] Romanos GE Javed F Romanos EB Williams RC. Oro-facial manifestations in patients with eating disorders. Appetite. (2012) 59:499–504. 10.1016/j.appet.2012.06.016 22750232

[29] Robinson L Micali N Misra M. Eating disorders and bone metabolism in women. Curr Opin Pediatr. (2017) 29:488–96. 10.1097/MOP.0000000000000508 28520584 PMC5675561

[30] Kavitha PR Vivek P Hegde AM. Eating disorders and their implications on oral health–role of dentists. J Clin Pediatr Dent. (2011) 36:155–60. 10.17796/jcpd.36.2.3785414p682843wj 22524077

[31] Striegel-Moore RH Dohm FA Kraemer HC Schreiber GB Taylor CB Daniels SR. Risk factors for binge-eating disorders: an exploratory study. Int J Eat Disord. (2007) 40:481–7. 10.1002/eat.20400 17573685

