# Supplementary material for: Is There an Association Between Fixed Orthodontic Treatment and Initiation of Eating Disorders? A Review of Currently Available Evidence
Source: Front Oral Health. 2021 Jul 12;2:707040. doi: 10.3389/froh.2021.707040 (PMC8757685; doi:10.3389/froh.2021.707040)
Supplement: Supplementary file 1 [file Table_1.DOCX]

**Supplementary Table A.** List of excluded studies with reasons after full-text review.

Alqahtani, H(Alqahtani, 2019) (review)

Neeley, W.W et al.(Neeley, et al., 2006) (review)

Shalish, M et al.(Shalish, et al., 2012) (no eating disorder)

Walter A. Bretz(Bretz, 2002) (review)

Marques LS et al.(Marques, Paiva, Vieira-Andrade, Pereira, & Ramos-Jorge, 2014) (no eating disorder)

Goldman, S.J.(Goldman, 2004) (review)

Mu Chen et al.(Chen, Wang, & Wu, 2010) (no eating disorder)

P. Colon et al.(Colon, Cougot, & Famery, 2018) (review)

Takakazu Yagi et al.(Yagi, et al., 2012) (review)

Tayer, B. H et al.(Tayer & Burek, 1981) (no eating disorder)

Patel, A. et al.(Patel, et al., 2009) (review)

Rosen, D.S. et al.(Rosen & Neumark-Sztainer, 1998) (review)

Abrams, R.A. et al.(Abrams & Ruff, 1986) (review)

Stege P. et al.(Stege, Visco-Dangler, & Rye, 1982) (review)

Montecchi, P. P. et al.(Montecchi, et al., 2003) (no orthodontic treatment)

Spigset, O.(Spigset, 1991) (no orthodontic treatment)

Roberts, M. W. et al.(Roberts & Li, 1987) (no orthodontic treatment)

Touyz SW et al.(Touyz, et al., 1993) (no orthodontic treatment)

Robb, N. D. et al.(Robb & Smith, 1996) (no orthodontic treatment)

Zachariasen, R. D.(Zachariasen, 1995) (review)

Milosevic, A.(Milosevic, 1999) (review)

Burden, D.(Burden, Mullally, & Sandler, 2001) (no eating disorders)

Faine MP(Faine, 2003) (review)

O'Reilly RL et al. (O'Reilly, et al., 1991) (orthodontic treatment not reported)
